# The association of demographic, psychological, social and activity factors with foot health in people with plantar heel pain

**DOI:** 10.1002/jfa2.70022

**Published:** 2024-12-11

**Authors:** Halime Gulle, Dylan Morrissey, Abdulhamit Tayfur, Dilber Karagozoglu Coskunsu, Stuart Miller, Aleksandra V. Birn‐Jeffery, Trevor Prior

**Affiliations:** ^1^ School of Physical Therapy and Rehabilitation Science Morsani College of Medicine University of South Florida Tampa Florida USA; ^2^ Sports and Exercise Medicine William Harvey Research Institute Bart's and the London School of Medicine and Dentistry Queen Mary University of London Mile End Hospital London UK; ^3^ Physiotherapy and Rehabilitation Faculty of Health Sciences Fenerbahce University Istanbul Turkey; ^4^ School of Sport, Rehabilitation and Exercise Science University of Essex Essex UK

**Keywords:** plantar fasciitis, psychosocial factors, quality of life

## Abstract

**Background:**

Plantar Heel Pain (PHP) can be a debilitating musculoskeletal condition from which only 50% recover within a year due to poor understanding of the mechanisms explaining severity and predicting outcomes specific to PHP.

**Objective:**

To explore associations between biopsychosocial variables and the severity of people with PHP. Secondly, to determine what combination of self‐reported factors distinguishes people with PHP from other foot pain (OFP).

**Methods:**

We collected data from 235 participants, including 135 (%57) PHP (age 44 ± 12 years, 66% female) and 99 OFP (%43) (age 38 ± 11 years, 57% female) using 5 demographic, 13 biomedical, 8 psychological, 3 social and 8 activity‐related factors. These were tested in linear and logistic regression models.

**Results:**

Quality of life (QoL) (*β* = 0.35; *p* < 0.001), education (*β* = −0.22; *p* = 0.003), gender (*β* = −0.20; *p* = 0.007), morning pain duration (*β* = −0.18; *p* = 0.01) and disease duration (*β* = −0.15; *p* = 0.040) were significantly associated with severity of PHP. The second model, without QoL, showed that having sensitisation (*β* = −0.18; *p* = 0.002) and a higher level of morning pain (*β* = −0.20; *p* = 0.01) are associated with severity. The logistic regression results revealed that people with PHP tend to have a systemic disease (OR = 3.34; 1.53–7.76), express more kinesiophobia (OR = 1.02; 1.01–1.14), are less likely to have previous injuries (OR = 0.40; 0.19–0.81), worse morning pain (OR = 1.02; 1.01–1.03) and standing pain (OR = 2.60; 1.39–4.87) compared to people with OFP.

**Conclusions:**

People with PHP have higher associated levels of a range of psychological, social and activity related factors than people with OFP. The findings highlight the importance of considering psychosocial assessments alongside physical examination.

## 1 BACKGROUND

Plantar Heel Pain (PHP) is one of the most common musculoskeletal conditions among adults. Plantar Heel Pain accounts for approximately 11%–15% of all foot complaints requiring professional care in adults and 8%–10% of all running‐related injuries [1, 2]. It is characterised by pain in the inferior‐medial regions of the rearfoot during weight‐bearing and is usually exacerbated by prolonged periods of standing and walking [3]. Hence, PHP can have a detrimental impact on health‐related Quality of life (QoL) due to the limited daily life activities of those affected [4].

There are a variety of management strategies for PHP, but the effectiveness is less than optimal [5, 6]. Current conservative management strategies include stretching, footwear modification, taping and patient education in first‐line management, with interventions such as shock wave therapy and orthoses increasingly available for those who fail to recover after first‐line management [7, 8, 9]. Lack of recovery could be because, nearly all previous observational studies designed to better understand PHP have focussed on physical impairments and biomedical factors. For instance, Hansen et al. (2019) focussed solely on biomedical factors like medical history, clinical symptoms and ultrasound examinations in their evaluation of the prognosis of PHP [10]. Similarly, Vertuccio et al. (2021) [11] and Fleischer et al. (2015) [12] were only focussed on demographics (such as age and gender) and clinical factors. In general, height, weight, Body mass index (BMI), age [13], decreased first MTPJ flexion [14], increased plantar fascia and heel pad thickness [15, 16] and decreased calf strength [17] have been found to be associated with PHP. However, better outcomes are not always linked to biomedical and physical mechanisms [18, 19]. While there is a substantial understanding of the relationship between biomechanical factors and PHP, Cotchett et al. (2020) [5] noted that people with PHP often report that their expectations and needs are frequently unmet. These unsatisfactory results may arise from the lack of tailored management strategies due to the limited understanding of the full range of biopsychosocial factors associated with PHP [20].

Psychosocial factors have been considered alongside physical factors in other musculoskeletal pain conditions [21, 22, 23]. A systematic review of low back pain treatment showed that patients with associated psychosocial problems who receive a psychosocial component in their rehabilitation were likely to experience less pain/disability than those receiving usual care [24]. While several observational studies have evaluated the biomedical factors [25, 26], and a few psychological variables linked to PHP [27, 28, 29], there is no research that has specifically evaluated the wide range of plausible biopsychosocial factors that are required to inform more nuanced intervention development.

The overarching aim of this study was to improve the understanding of PHP by constructing explanatory models from a wide range of self‐reported biopsychosocial factors to understand better the severity of PHP and, secondly, explore what combination of self‐reported factors distinguish people with PHP from other foot pain (OFP).

## 2 METHODS

This is an international case‐control study nested within a cohort study [30, 31] to investigate associations between biopsychosocial variables and severity of people with PHP and to identify which combination of self‐reported factors differentiates individuals with PHP from those with OFP. The study procedures were approved by XXX Research Committee (approval No. QMREC2018/92) and XXX Research Ethics Committee (approval No: 264615). Electronic informed consent was sought from each recruited participant prior to the completion of the online questionnaire. The STROBE (**St**rengthening the **R**eporting of **Ob**servational Studies in **E**pidemiology) statement was followed as a guideline for the design and reporting of this study (supplementary Table 1).

### 2.1 Participants and screening process

Participants were recruited in Turkey, the UK, France and Spain via advertising in hospitals and physiotherapy clinics, posters in public areas and social media outlets. The inclusion criteria were having a clinical diagnosis of PHP or another clinically diagnosed ankle or foot musculoskeletal condition within the last 6 months. The majority of participants (%72) were diagnosed by a podiatrist with over 30 years of clinical experience and an orthopaedics based on reported symptoms and clinical examination. Participants with early morning and first step pain for more than 1 month and pain on palpation of the plantar medial tubercle of the calcaneus were classified as people with PHP compared to other foot problems. The rest of the sample were recruited by GPs and physiotherapists from other clinics (10%), a consultant physiotherapist (%13) and social media (5%). Additionally, six further questions were asked to confirm the diagnosis of participants in the questionnaire battery. The questions were: *(1) Please describe your main problem?; (2) What was your diagnosis in right/left foot?; (3) Who diagnosed your condition?; (4) Which investigations did you have for your conditions?; (5) How many visits have you made to the clinician for your problem?* and *(6) How long have you had this condition?* Participants who did not provide diagnostic details and medical history were excluded from the study. People under 18 years of age were not eligible to join the study.

### 2.2 Measures

The data collection was completed using an online survey, which was constructed and administered using ‘SmartTrial’ https://www.smart‐trial.com. The validity and reliability of this online questionnaire battery were evaluated and established with a previously published feasibility study [32] prior to implementation. Translation, cross‐cultural adaptation and validation of self‐reported outcome measures in different languages were identified and integrated into this paper from the literature. The online survey also included the pain map to assess the area and distribution of pain, namely the Navigate Pain app (version 1.0; Aalborg University, Denmark) [33, 34].

#### 2.2.1 Main patient reported outcome measure

The Foot Health Status Questionnaire (FHSQ) was selected as the main outcome measure due to having high responsiveness to the degree of participants' PHP [35] and to follow recommendations from the relevant guidelines [3]. It comprises 13 questions, categorised into pain, function, footwear and general foot health; and uses a 5‐point Likert scale across four subscales [36]. For each subscale, the total score ranges from 0 to 100 points, with 0 representing the worst foot health and 100 best [37]. The FHSQ subscales have demonstrated high test‐retest reliability, content, construct and criterion validity [36].

#### 2.2.2 Health related quality of life

The Euro QoL (Euroqol), 5 dimensions, 5 level questionnaire (EQ‐5D‐5L) measures overall health related QoL in five dimensions; mobility, self‐care, usual activities, pain/discomfort and anxiety/depression. The responses can be converted into a single preference‐based index anchored on a scale where −1 and 1 represent being ‘dead’ and full health, respectively [38].

#### 2.2.3 Biomedical measures

A range of characteristics was recorded including medical history, duration of symptoms, side effects (left, right, or bilateral) and the duration/severity of pain beneath the heel over the previous week. In the FHSQ, comorbidity was defined as any medical condition reported by a participant for which she or he was taking medication.

#### 2.2.4 Psychological measures

The Pain Catastrophizing Scale (PCS) was used to measure pain‐related catastrophizing [39]. It has 13 items that yield three subscale scores (rumination, magnification and helplessness) and an overall score, with higher total scores indicating more catastrophic behaviour [40]. Reliability and validity of the PCS have been established [41].

The Fear‐Avoidance Belief Questionnaire (FABQ) is designed to assess fear of avoidance beliefs on movement for use in patients with musculoskeletal conditions and chronic pain [42]. Items are scored on a seven‐point Likert scale, with higher values indicating greater fear of movement. The FABQ demonstrates high levels of internal consistency and test‐retest reliability [43, 44], therefore a useful screening tool for identifying patients at risk of a poor outcome [45].

The Central Sensitisation Inventory (CSI) is a 25‐item questionnaire (two parts) developed to detect central sensitisation symptoms in clinical settings. The CSI has high levels of internal consistency and test‐retest reliability [46].

Additionally, we considered that participants' beliefs about their prognosis, or future condition, may be associated with severity; hence three questions were prepared to understand patients' future beliefs: *1) Do you think your condition will be better/worse/no change?; 2) How confident are you with this recovery prediction?; 3) Please predict how long this recovery will take?;* with follow‐up questions of *Why do you think you will get worse?* (those who are selected will be worse than Q1); *Why do you think you will not improve?* (those who are selected will not change to Q2).

#### 2.2.5 Social factors

Measures of occupation, education and ability to readily use information technology were collected. The occupational category combined information on occupation and employment status and yielded six separate classifications: white‐collar professional, white‐collar other, blue‐collar, retired, homemaker and other [47]. Classification of education status was based on information about the highest education level completed. From this standard, the following categories were created: did not attend, primary school, secondary school, college/high school, bachelor, master's and PhD. To assess participants' perceived skills in using information technology for health, we used the eHealth Literacy Scale (eHEALS), which consists of eight 5‐point Likert scales (1‐strongly disagree to 5‐strongly agree), with the total ranging from 8 to 40; a higher score indicating higher literacy. Reliability and validity of the eHEALS have been confirmed [48].

#### 2.2.6 Activity related measures

The Global Physical Activity Questionnaire (GPAQ) comprises 16 items that measure physical activity in work, transport, leisure activities and time spent inactive and covers several components of physical activity (intensity, duration and frequency). The unit of GPAQ is MET, which is defined as the energy cost of sitting quietly and is equivalent to a caloric consumption of 1 kcal/kg/hour. The GPAQ showed acceptable evidence of short‐ and long‐term test‐retest reliability by activity category and modest validity evidence [49]. Additionally, hours standing was measured with a specific question: “How much time do you spend on your feet in a typical day?” Answers were recorded as hours and minutes. Specific questions relating to sports participation, running history including weekly running mileage, participation frequency and training surface were also constructed.

### 2.3 Data analysis

Height and weight measures were expressed as centimetres and kilograms, from which BMI was calculated (kg/cm^2^). Categorical and ordinal data were electronically transcribed from SmartTrial then recoded for statistical calculations in STATA (version 16.0, StataCorp LP, College Station, TX, USA). Categories within the comorbidity, education and ethnicity variables were combined to eliminate sparseness retaining a ratio of ≥20 participants per estimated model parameter. We treated a categorical factor (disease duration) as continuous if linearity with outcome could be assumed after visual examination using scatter plots. Missing values were not imputed and models were developed only from participants with complete data.

To assess the area and distribution of pain, the total area drawn expressed as the total number of pixels was extracted for each pain map. The Navigate Pain system also provided average, usual and current pain level for each drawing. Further, the total number of independent non‐contiguous pain sites was manually recorded.

### 2.4 Statistical analysis

Group data were reported as mean (SD) and frequency count (%) as appropriate. All analyses were performed using STATA (version 16.0, StataCorp LP, College Station, TX, USA). All variables were explored for normality by inspection of histograms and de‐trended Q‐Q plots and checked for skewness and kurtosis prior to statistical analysis. To compare the PHP and OFP groups, continuous data were assessed with a one‐way ANOVA; ordinal and categorical data were assessed with chi‐square; and differences were described using effect size measures with Cohen's d for continuous variables and Cramér's V for categorical variables [50].

Multivariable linear regression was used to develop a model of PHP severity with the FHSQ general foot health subscale as the dependent variable. To facilitate variable selection, we used univariate analyses to assess crude associations with correlation coefficients (the significance level was set at *p* < 0.01). A final model was developed hierarchically by manually entering significant variables from the univariate analysis and comparing models using the likelihood ratio test.

For the second aim, we built a logistic regression model using univariate analyses to assess crude associations between variables and conditions (0 = OFP and 1 = PHP). The same model building approach was used for the multivariable linear regression. Model fit was tested with Hosmer‐Lemeshow. Accuracy, specificity and sensitivity of the model were also assessed. Prior to multivariable linear and logistic regression, correlations between explanatory variables were evaluated to detect levels of association and avoid issues relating to multi‐collinearity by calculating variance inflation factors (VIFs). The level of collinearity was considered problematic and one of the two independent variables not included in the model if the mean VIF was ≥5 and individual VIFs were ≥10 [51]. (Collinearity analysis results are presented in the supplementary table 2).

## 3 RESULTS

### 3.1 Sample characteristics

**FIGURE 1:**
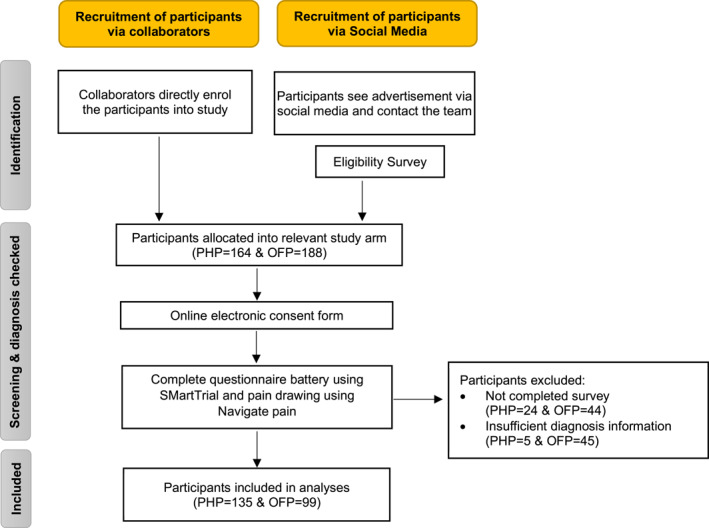
Participant enrolment and screening process. OFP = other foot pain; PHP = plantar heel pain.

Two hundred and thirty four people participated in the study. Among them, 135 participants (57%) had PHP with an average age of 44 ± 12 years, 65% female, a BMI of 26 ± 4 and weekly activity levels (expressed in metabolic equivalents task (MET)‐minutes) of 5393 ± 6557. Additionally, 99 participants (43%) had OFP with an average age of 38 ± 11 years, 54% female, a BMI of 25 ± 4 and weekly activity levels of 5498 ± 6983. The participants were recruited from the UK (*n* = 121), Turkey (*n* = 92), Spain (*n* = 4) and France (*n* = 17) through advertising in hospitals and physiotherapy clinics, posters in public areas and social media outlets over a year. The diseases that constituted OFP are Achilles tendinopathy (*n* = 34), tibialis posterior tendinopathy (*n* = 25), ankle sprain (*n* = 28) and peroneal tendinopathy (*n* = 12). Worst pain over the last week for the PHP and OFP groups was 29 ± 2 and 25 ± 3 on a 100‐point scale, respectively. All participants deemed eligible (Figure 1) completed all outcome measurements online without any missing data. There was a statistically significant difference between groups regarding all psychological factors apart from depression (Table 1). No between‐group mean differences were found for activity related factors. All biopsychosocial variables are presented in Table 1.

**TABLE 1:** Population characteristics and groups comparison between people Plantar Heel Pain (PHP) and other foot pain (OFP).

Population characteristics			
Variables	PHP (*n* = 135)	OFP (*n* = 99)	
	Mean ± SD or *n* (%)	Mean ± SD or *n* (%)	Effect size
Quality of life EQ5D5L‐index, (0–1)	^§^0.67 ± 0.2	0.76 ± 0.1	0.41
**Demographics**			
Age, years	^§^44.1 ± 12.1	38.1 ± 11.5	−0.47
BMI, kg/m^2^	^§^26.9 ± 4.4	25.1 ± 4.6	−0.38
Sex, (female: Male)	88:47	53:46	0.11
Ethnicity (white: Asian: Other: PNTS)	98:26:4:7	69:14:6:10	0.14
Dominant leg (right: left: not sure)	112:16:8	79:14:6	0.04
**Biomedical**			
General foot health, FHSQ, (0–100)	^§^35.1 ± 25	49.1 ± 24	0.57
Foot pain, FHSQ, (0–100)	^§^49.9 ± 24	64.9 ± 22	0.62
Foot function, FHSQ, (0–100)	^§^56.2 ± 30	74.3 ± 24	0.64
Morning pain duration, mins.	29.4 ± 67	25.8 ± 64	−0.54
Morning pain severity, VAS	^§^58.4 ± 25	42.2 ± 23	−0.65
Disease duration			
0–6 months	37 (28%)	31 (32%)	
6–12 months	18 (13%)	16 (16%)	
1–2 years	29 (21%)	14 (14%)	0.18
2–3 years	18 (13%)	12 (12%)	
More than 3 years	33 (25%)	26 (26%)	
Onset of pain (sudden: Gradual: Other)	92:40:3	98:1:0	0.39
Co‐morbidities			
MSK (back pain, osteoarthritis, RA)	24 (18%)	7 (7%)	
Systemic (cholesterol. Diabetes, HT, HD, LD)	48 (35%)	9 (9%)	
Psychological disease (depression, anxiety)	23 (18%)	11 (11%)	0.44
None	40 (29%)	72 (73%)	
Number of co‐morbidities	^§^1.3 ± 0.7	1.1 ± 0.4	−0.37
Back pain presence, *n* (%)			
Yes (current, recurrent)	66 (49%)	39 (39%)	
Yes (previously)	38 (28%)	27 (27%)	0.13
No	31 (23%)	33 (34%)	
Back pain spreading to;			
Thigh and knee, *n* (%)	17 (41%)	12 (50%)	
Shank, *n* (%)	6 (14%)	2 (8%)	0.10
Foot, *n* (%)	18 (43%)	10 (41%)	
Back pain association with leg pain, yes, *n* (%)	41 (39%)	24 (36%)	−0.03
First symptoms noticed (pain: Stiffness: Swelling: Other)	125:7:3:1	84:8:4:3	0.12
Pain in walking (worse: Better: no change)	76:48:12	35:43:21	0.23
Pain in standing (worse: Better: no change)	103:7:26	49:10:40	0.26
Pain in sitting (worse: Better: no change)	40:65:31	15:54:30	0.17
Having previous injury (yes: no)	43:93	42:57	0.10
Investigation types (ultrasound: Physical examination: MRI: xRAY: Blood tests: Other)	38:52:12:15:3:16	24:28:6:8:30:3	0.42
Number of investigations	5.9 ± 5.3	4.6 ± 6.3	−0.25
Number of visits to health professional	^§^1.3 ± 0.7	1.1 ± 0.4	−0.22
Sleeping duration, hours	6.9 ± 1.1	7.2 ± 1.0	0.26
Sleeping difficulties (yes: no)	78:57	23:76	0.20
Reason sleep difficulties (foot pain: Other pain: Depression: Anxiety: Other)	33:13:27:2:1	6:2:2:7:6	0.65
Feeling rested (yes: Partially: No)	32:75:28	35:52:12	0.13
Smoking			
Yes (active, social smokers)	24(18%)	17 (17%)	
No (passive, ex‐smokers)	46(34%)	25 (25%)	0.10
Never smoked	65(47%)	57 (56%)	
Family history (Tendon disorders: Systemic disease –Psoriasis & AS & RA: None: Other)	7:27:89:12	7:20:68:4	0.75
**Psychological**			
Catastrophization, PCS (0–52)	^§^15.0 ± 12.3	9.6 ± 9.6	−0.48
Sensitisation, CSI, (0–100)	^§^32.2 ± 17	26.4 ± 14.5	−0.35
Fear avoidance‐ work, FABQ	^§^10.5 ± 9.7	7.1 ± 8.5	−0.47
Fear avoidance‐ PA, FABQ	^§^14.2 ± 5.6	11.5 ± 5.5	−0.37
Depression diagnosis, (yes: no)	11:124	5:95	0.06
Condition prediction (get better: get worse: no change: don't know)	63:21:12:39	53:16:11:19	0.11
Condition confidence, (out of 100)	77.2 ± 20.0	84.4 ± 0.5	0.33
Time prediction, months	73.1 ± 5.3	76.4 ± 6.3	0.13
**Social**			
Educational level, *n* (%)			
Elementary school	16 (12%)	4 (4%)	
High school	26 (19%)	15 (15%)	
Bachelor	62 (46%)	50 (50%)	0.22
Master's degree	20 (15%)	26 (26%)	
PhD	11 (8%)	4 (4%)	
Occupation *n* (%)			
Blue‐collar	17 (12%)	7 (7%)	
White collar	54 (40%)	61 (62%)	
Professionals & athlete	22 (16%)	15 (15%)	0.34
Unemployment & students	20 (15%)	12 (12%)	
Homemakers & retired	22(16%)	4 (4%)	
Health literacy, eHEALS	28 ± 6	28 ± 6	0.16
**Activity**			
Activity level, GPAQ	5363 ± 6187	5498 ± 6983	0.12
Hours standing	6.5 ± 3	6.1 ± 3	−0.01
Sports participation, yes *n* (%)	57(42%)	64(64%)	−0.22
Type of sports (running: Yoga: Football: Basketball & others)	21:19:9:8	1:28:9:22	0.54
Number of sports participated in	0.64 ± 0.8	1.46 ± 0.7	1.00
Sports age, years	14.3 ± 14.2	13.2 ± 10.3	−.08
Footwear, FHSQ footwear	^§*^51.9 ± 25	43.1 ± 26	−0.34
Running distance	18.1 ± 13.1	21.3 ± 15.0	0.22
**Pain map (PHP:57, OP:46—*n* = 103 in total)**			
Total area drawn (pixel number)	3870 ± 3081	4108 ± 3854	0.11
Current pain level (out of 10)	5.30 ± 2.59	4.62 ± 2.91	−0.34
Usual pain level (out of 10)	5.12 ± 2.84	4.42 ± 2.57	−0.13
Total number of painful sites	3.53 ± 2.65	3.20 ± 2.19	0.08

*Note*: Results are given in two groups to demonstrate differences as mean ± SD or total number with percentage in the group. Effect size measured with Cohen's d for continuous variables and Cramér's V for categorical variables. Key: SD=Standard deviation of mean values; *n* = Number of participants; PHP: Plantar heel pain; OFP = other foot pain, BMI=Body Mass Index; PNTS= Prefer not to say; min = minutes; VAS = visual analogue scale; EQ5D5L = The Euro quality of life (Euroqol) five dimension five level; MSK = Musculoskeletal; AS = Ankylosing spondylitis RA = Rheumatoid arthritis; HT= Hypertension; HD= Heart diseases; LD = Lung disease; GPAQ = Global Physical activity questionnaire; FHSQ= Foot Health Status Questionnaire; PCS: Pain Catastrophization Scale; CSI: Central Sensitization Inventory; FABQ: Fear avoidance behaviour; Global Physical Activity Questionnaire; §*p* < .05 compared to other foot pain.

### 3.2 Multivariable linear regression to explain Plantar Heel Pain severity

Univariate correlations between a range of biopsychosocial factors and foot health (general foot health subscale of FHSQ) were found within the PHP group with univariate analyses (supplementary Table 3). Quality of life, sensitisation and catastrophisation showed the largest correlations (*r* ^2^ = 0.15, *r* ^2^ = 0.10 and *r* ^2^ = 0.09, respectively). In the social subgroup of variables, the only statistically significant correlation was with education (*r* ^2^ = 0.07). All univariate analysis results are reported in supplementary Table 3.

The multivariable regression for severity revealed that QoL (β = 35.4, 95% CI, 19.4–51.4), education [β (95% CI), −17.8 (−29.3 to −6.3)], gender [β (95% CI), −11.1 (−19.1 to −3.1)], disease duration [β (95% CI), −1.8 (−3.5 to −0.8)] and morning pain duration [−0.07 (−0.13 to −0.01)] were the only constructs significantly associated with the overall severity of PHP measured by general foot health; meaning higher PHP severity was associated with lower QoL, lower education level, being female, longer morning pain and longer disease duration (Table 2). The model [F (5,129) 10.94, *p* ≤ 0.001] explained 29% of the total variance.

**TABLE 2:** Multivariable/univariate linear regression analysis for condition severity of people with Plantar Heel Pain (PHP) (*n* = 135).

Variables	Univariate analysis	Multivariable analysis (*R* ^2^ = 0.29 Adjusted *R* ^2^ = 0.27)		
	Coef. (95% CI)	Coef. (95% CI)	β coef.	*p* value
Higher quality of life, EQ5D5L‐index	41.5 (24.5–58.3)	35.4 (19.4–51.4)	0.35	<0.001
**Social**				
Stopping education earlier	−21.1 (−34.2 to −7.8)	−17.8 (−29.3 to −6.3)	−0.22	0.003
**Biomedical**				
Being female	−10.9 (−19.8 to −2.1)	−11.0 (−19.1 to −3.1)	−0.20	0.007
Longer morning pain duration, mins.	−0.06 (−0.13 to 0.001)	−0.07 (−0.13 to −0.01)	−0.18	0.01
^‡^Longer PHP duration, years	−2.5 (−4.5 to −0.6)	−1.8 (−3.5 to 0.08)	−0.15	0.04

*Note*: *R* ^2^: statistical measure that represents the proportion of the variance for a dependent variable that's explained by an independent variable or variables in a regression model. The dependent variable is general foot health subscale of FHSQ, which is 0‐100 scale, indicating worse to better foot health score. Negative coefficients mean an increased severity of PHP condition, while positive coefficients mean a decreased severity of PHP condition. Key: mins = minutes, CI: Confidence Interval, *β* = Beta, Coef = coefficient. ‡ Handled as continuous in the models using the combined categories, assuming linearity and the coefficients are per category increase.

The second multivariable regression for severity, performed with the EQ‐5D‐5L removed demonstrated that gender [β (95% CI), −8.16 (−19.1 to −3.1)], morning pain duration [−0.06 (−0.13 to −0.01)], morning pain severity [β (95% CI) −0.17 (−3.5‐0.08)], education [β (95% CI), −16.3 (−29.3 to −6.3)] and sensitisation [β (95% CI), −0.27 (−3.17 to 0.16)] were the only constructs significantly associated with the overall severity of PHP measured by general foot health; meaning higher PHP severity was associated with being female, longer morning pain duration and severity, lower education level, higher sensitisation (Table 3). The model [F (5,129) 10.94, *p* ≤ 0.001] explained 23% of the total variance.

**TABLE 3:** Multivariable/univariate linear regression analysis for condition severity of people with Plantar Heel Pain (PHP) (*n* = 135).

Variables	Univariate analysis	Multivariable analysis (*R* ^2^ = 0.23 Adjusted *R* ^2^ = 0.20		
	Coef. (95% CI)	Coef. (95% CI)	β coef.	*p* value
**Biomedical**				
Being female	−10.9 (−19.8 to −2.1)	−8.67 (−19.1 to −3.1)	−0.16	0.05
Longer morning pain duration, mins.	−0.06 (−0.13 to 0.001)	−0.06 (−0.13 to −0.01)	−0.17	0.03
^‡^Longer morning pain severity, (0–100)	−2.5 (−4.5 to −0.6)	−0.17 (−3.5 to 0.08)	−0.20	0.01
**Social**				
Stopping education earlier	−21.1 (−34.2 to −7.8)	−16.34 (−29.3 to −6.3)	−0.20	0.01
**Psychology**				
Sensitisation	0.10 (−0.31 to 0.11)	−0.27 (−3.17 to 0.16)	−0.18	0.02

*Note*: *R* ^2^: statistical measure that represents the proportion of the variance for a dependent variable that's explained by an independent variable or variables in a regression model. The dependent variable is general foot health subscale of FHSQ, which is 0‐100 scale, indicating worse to better foot health score. Negative coefficients mean an increased severity of PHP condition, while positive coefficients mean a decreased severity of PHP condition. Key: mins = minutes, CI: Confidence Interval, *β* = Beta, Coef = coefficient. ‡ Handled as continuous in the models using the combined categories, assuming linearity and the coefficients are per category increase.

### 3.3 Multivariable logistic regression comparing people with Plantar Heel Pain and other foot pain

In univariate analyses, people with PHP were older (OR: 1.03; 95% CI, 1.01–1.06) and had a higher BMI (OR: 1.09; 95% CI, 1.02–1.16), compared to people with OFP. The PHP group had greater levels of psychological conditions (OR = 1.02–1.08; 95% CI, 1.01–1.14). Similarly, there were notably different biomedical factors (i.e., age, BMI, gender, number of comorbidities and pain during standing or walking). All univariate analyses results are presented in supplementary Table 4.

A model with five independent variables including having systemic disease, degree of fear avoidance, morning pain severity, having pain during standing and having unilateral pain accounted for 21% of the variance in the presence of PHP. The results reveal that, compared to people with OFP, people with PHP tend to have a systematic disease (OR = 3.34; 95% CI, 1.53–7.76), express more fear avoidance (OR = 1.02; 95% CI, 1.01–1.14), have worse morning pain (OR = 1.02; 95% CI, 1.01–1.03) and worse pain when standing (OR = 2.60; 95% CI, 1.39–4.87) but were less likely to have a unilateral previous injury (OR = 0.40; 95% CI, 0.19–0.81) (Table 4). Model fit was good (Hosmer‐Lemeshow test = 0.75, *p* < 0.001) with acceptable accuracy (AUC = 0.78), specificity (69.8%) and sensitivity (70.1%).

**TABLE 4:** Multivariable/univariate logistic regression analysis by comparing people with Plantar Heel Pain (PHP) (*n* = 135) and people with other foot pain (OFP) (*n* = 99).

Model_differentiation_ (Sensitivity = 0.70, specificity = 0.69, AUC = 0.78)					
Variables	Univariate analyse		Multivariable analyse		
	Odd ratio	95% CI	Odds ratio	95% CI	*p* value
**Biomedical**					
Severe morning pain	1.02	1.01–1.03	1.02	1.01–1.03	<0.001
Having pain during standing	3.15	1.80–5.50	2.60	1.39–4.87	0.003
Having a systemic disease	3.74	1.76–7.93	3.34	1.53–7.76	0.005
Having unilateral previous injury	0.49	0.27–0.91	0.40	0.19–0.81	0.01
**Psychological**					
More fear avoidance behaviour	1.04	1.01–1.06	1.02	1.01–1.04	0.03

*Note*: The dependent variable is having PHP versus having other foot and ankle related musculoskeletal conditions. Odd ratios were the likelihood of having PHP, meaning greater than one increases the possibility of having PHP, while less than 1 decreases the possibility of having PHP. Key: mins = minutes, CI: Confidence Interval, AUC = area under the curve.

## 4 DISCUSSION

### 4.1 Severity of Plantar Heel Pain

The primary aim of this study was to evaluate the association between self‐reported biopsychosocial factors with the severity of PHP. Multivariable regression revealed that QoL, education, gender, disease duration and morning pain duration were significantly associated with the overall severity of PHP. The second model, without general QoL, showed that having pain sensitisation and a higher level of morning pain are associated with PHP severity in a model including gender, education and longer morning pain duration. This dual approach shows that three factors (gender, morning pain duration and educational level) are robustly associated with severity, while global QoL measures, condition duration, degree of pain and pain sensitisation have a less clear but nonetheless meaningful relationship to severity. These findings also indicate that BMI is not related to the severity of PHP, which contradicts previous literature [25]. All of these factors should be included in assessment of patients with PHP in usual care where it is feasible to do so and strongly considered in future research, while the clinical community needs to reconsider the role of BMI.

Quality of life, education, gender, disease duration and morning pain duration were associated with the severity of PHP in the multivariable model. The strongest associations with the severity of PHP were QoL score, in the multivariable model which included for education, gender and morning pain and symptom duration. Given the broad impact of pain on the enjoyment of life in general, emotional well‐being, fatigue and weakness [52, 53], the result is not surprising, but supports the importance of assessing QoL in people with PHP. It should also be noted that the QoL measured by EQ5D5L covers multi‐aspect of wellbeing such as pain, function and daily activities and psychological conditions. Therefore, EQ5D5L could be a useful assessment to help explain and understand a patient's presentation including the psychological aspect.

The second model's results showed that sensitisation is one of the associated factors related to PHP severity. Several studies have explored sensitisation in this population Fernández‐Lao et al. (2016) discovered that individuals with PHP exhibited widespread pressure pain hyperalgesia in distant pain‐free areas compared to healthy individuals, indicating altered central pain processing in this condition [54]. Similarly, Plaza‐Manzano et al. (2019) suggested that individuals with unilateral chronic PHP exhibit widespread pressure hypersensitivity over both nerve trunks and musculoskeletal structures [55]. Wheeler (2019) found that people with chronic PHP have higher central sensitisation scores compared to those with other tendinopathies [56]. Therefore, regardless of its peripheral presentation, clinicians should not rule out the possibility of altered central pain processing in patients with PHP. More research into the pain input and processing mechanisms associated with PHP is warranted.

Education level was the second most significant correlate in this sample, with lower education level being associated with poorer foot health in people with PHP when controlling for QoL, gender, morning pain and symptom duration. One explanation for this, as hypothesised by Kamaleri et al. (2009) [57], suggests that individuals who leave school during their elementary or junior‐high years may be likely to find employment in manual labour positions. This requires physical demand, which is a predisposing factor for PHP [58]. Moreover, individuals with lower education levels are more likely to have lower incomes, which can impact their access to better healthcare. In addition, individuals with lower education levels are more likely to have difficulties with the most fundamental school‐based knowledge. Given that a recent best practice guide has identified that education about the condition is an important part of first line management [7], an awareness of the level of education may be of use to clinicians.

The findings in this study are in line with a similar study [27] that found being female explained 29% of foot health scores in total, beyond a model including QoL, education, morning pain and symptom duration. Several studies reported that it is well established that gender differences in pain and function exist, but the reason for the association is still unknown [59]. It has been suggested that an interaction of biological, psychological and sociocultural factors likely contributes to these differences [60]. Therefore, further research exploring the association between gender and the severity of PHP is needed.

The significant relationship between morning pain and foot health in this sample, indicated that longer and higher pain in the morning was associated with poorer foot health in people with PHP after controlling for QoL, gender and education. Morning pain and stiffness are significant factors in diagnosing PHP [61]. Similarly, patients with longer duration, more severe symptoms are less likely to respond to treatment and have an increased likelihood of chronicity due to changes in peripheral pain processing and psychological responses to pain [62, 63]. Our study findings are consistent with those reported by Klein et al. (2012), indicating that symptoms of plantar fasciitis persist for more than 6 months and patients do not experience an improvement in pain intensity or functional limitations [64].

### 4.2 Comparison between people with Plantar Heel Pain and other foot pain

For the second aim of determining how people with PFP differ from those with OFP, we found that those with PHP have higher levels of biomedical and psychological impairments such as severe morning pain, systemic disease presence, pain during standing and fear avoidance, than people with other foot problems.

Understanding the differences between people with PHP and OFP is important because, whilst they may present with similar symptoms, they may require different assessment and management techniques to optimise outcomes. When we compared PHP with OFP, severe morning pain and an increase in pain during prolonged standing beneath the heel tended to indicate PHP, when controlling for systemic disease, unilateral previous injury and fear avoidance behaviour. Findings provide additional support for the typical presentation of PHP [65, 66].

After controlling for morning pain, pain during standing, systemic disease and fear avoidance, a previous unilateral injury was significantly less likely to indicate PHP than OFP (odds ratio = 0.49). This reflects the typical presentation of PHP being of insidious onset. Additionally, OFP can develop when gait is changed suddenly due to the quick onset of pain from an injury. Such a change in gait can result in a range of OFP either ipsilaterally or contralaterally. Therefore, a history of unilateral previous injury could be associated with any type of foot pain and is rarely associated with PHP onset.

Having a systemic disease had the highest odds ratio for distinguishing PHP and OFP in this sample, indicating that people with PHP are more likely to have a systemic disease compared to OFP. These systemic diseases include seronegative arthritis, psoriatic arthritis, diffuse idiopathic skeletal hyperostosis, rheumatoid arthritis, fibromyalgia and gout [65]. Rogers et al. (2021) [67] found that factors such as waist girth, ankle plantar flexor strength, multisite pain and pain catastrophizing were independently linked to chronic PHP, indicating central or systemic associations, rather than foot‐specific factors. Similarly, Thomas et al. (2010) [68], Rio et al. (2015) [69] and Lui (2010) [70] reported that various systemic diseases can be related to presentation of heel pain. However, determining an exact aetiology is often difficult [1]. Therefore, in the process of taking a history and conducting a physical examination, the clinician should consider systemic symptoms and concomitant arthralgias to optimise diagnostic and therapeutic success.

An association between kinesiophobia and PHP has previously been identified [29]. A recent meta‐analysis and a cross‐sectional study found a moderate positive relationship between kinesiophobia and disability in people with PHP [29, 71]. Further, kinesiophobia was not significantly associated with pain severity in other populations [72, 73]. Consequently, this would indicate that assessment and management should be based on a range of symptoms, medical history and the psychological aspect of the disease.

### 4.3 Strengths, limitations and future directions

The main study limitation was the absence of some clinical and radiological examinations. An evaluation of the model suggests that variables not included in this study might influence the severity of PHP, including biomechanical variables such as variations in foot posture [74] and imaging findings such as the thickness of the plantar fascia [75] or radiographic evidence of a calcaneal spur [75]. The addition of other clinical or imaging variables to the current model may alter the significance of the associations identified in this study to PHP. Another limitation might be the lack of structured training for the clinicians who diagnosed the participants, although we provided them with diagnostic guidance. Finally, due to study design, establishing causal relationships and the directionality of associations between variables is not appropriate.

Despite the limitations of this study, the strengths include the large international sample of people with PHP from a broad general public sample; assessment of a broad range of biomedical, psychological, social and activity domains of health; and the deployment of an accessible and easy to administer self‐reported set of widely used measures. An important consideration before interpreting the results of the present study was the extent to which our participants could be considered representative of the population. For both groups, the level of pain [74, 76, 77], duration of symptoms [76, 77, 78], BMI [74, 76, 77, 78], age [74, 76, 77, 78] and percentage of females [74, 76] were similar to other studies that have evaluated risk factors and interventions for PHP and OFP.

There are several potential avenues for further research into the biopsychosocial features of PHP. One involves the study of biomechanical factors pertaining to kinetic, kinematic and neuromusculoskeletal impairment of PHP. These factors have been found to influence the experience of musculoskeletal pain and will add further depth to our understanding of PHP subgroups. A second research approach could investigate the causal aspects of these factors in PHP. This would require prospective cohort studies that are more suited to the validation of temporal relationships. If high levels of symptom severity are an indicator of biopsychosocial problems, then early intervention aiming to reduce the severity of PHP may prevent the development of chronicity and reduce impact on overall well‐being. Thus, a third research direction could explore the prognostic capabilities of biopsychosocial factors in PHP and how attending to these might impact on treatment outcomes.

## 5 CONCLUSION

The self‐reported biopsychosocial variables related to PHP severity include QoL, education, gender, morning pain duration and disease duration. These findings show that severity of PHP is more than just a mechanical or biomedical problem. Diverse psychological, social and activity‐related factors are present and influence foot health. Additionally, those with PHP have higher levels of biomedical and psychological impairments such as severe morning pain, systemic disease, standing pain and fear avoidance than people with other foot problems. Although causality cannot be determined in this study and the relations among these variables are not fully understood, this information may be helpful in optimising PHP management. Clinicians should therefore consider the presence and potential role of these variables in the overall care of their patients. Prospective cohort studies are needed to confirm these associations and establish causal and temporal relationships with outcomes.

## AUTHOR CONTRIBUTION

**Halime Gulle:** Conceptualization; data curation; formal analysis; investigation; methodology; project administration; resources; software; validation; visualization; writing—original draft; writing—review and editing. **Dylan Morrissey:** Supervision; conceptualization; data curation; investigation; methodology; project administration; resources; software; validation; visualization; writing—review and editing. **Abdulhamit Tayfur:** Data curation; formal analysis; investigation; methodology; software; validation; visualization; writing—review and editing. **Dilber Karagozoglu Coskunsu:** Data curation; formal analysis; investigation; methodology; resources; validation; writing—review and editing. **Stuart Miller:** Formal analysis; Investigation; methodology; visualization; writing—review and editing. **Alexandra V. Birn‐Jeffery:** Supervision; conceptualization; data curation; investigation; methodology; project administration; software; writing—review and editing. **Trevor Prior:** Supervision; conceptualization; resources; investigation; project administration; writing—review and editing.

## CONFLICT OF INTEREST STATEMENT

The authors declare no conflicts of interest.

## ETHICS STATEMENT

Ethics approval for the current study was obtained from ‘Queen Mary Ethics of Research Committee’ on May 15, 2019 (approval No. QMREC2018/92), ‘National Health Service ethics—London City & East Research Ethics Committee’ on September 10, 2019 (approval No: 264615), University of Liège Hospital‐Faculty Ethics Committee (2019/182) and by the Medipol University Non‐Interventional Clinical Research Ethics Committee (2020/31) Electronic informed consent was sought from each recruited participant prior to completion of the online questionnaire.

## CONSENT FOR PUBLICATION

Not applicable.

## Supporting information

Supplementary Material

## Data Availability

The datasets analysed during the current study are available from the corresponding author on reasonable request.

## References

[1] Martin, Robroy L. , Todd E. Davenport , Stephen F. Reischl , Thomas G. McPoil , James W. Matheson , Dane K. Wukich , Christine M. McDonough , et al. 2014. “Heel Pain—Plantar Fasciitis: Revision 2014.” Journal of Orthopaedic & Sports Physical Therapy 44(11): A1–A33. 10.2519/jospt.2014.0303. 25361863

[2] Taunton, J. E. , M. B. Ryan , D. B. Clement , D. C. McKenzie , D. R. Lloyd‐Smith and B. D. Zumbo . 2002. “A Retrospective Case‐Control Analysis of 2002 Running Injuries.” British Journal of Sports Medicine 36(2): 95–101. 10.1136/bjsm.36.2.95. 11916889 PMC1724490

[3] McPoil, Thomas G. , RobRoy L. Martin , Mark W. Cornwall , Dane K. Wukich , James J. Irrgang and Joseph J. Godges . 2008. “Heel Pain—Plantar Fasciitis.” Journal of Orthopaedic & Sports Physical Therapy 38(4): A1–A18. 10.2519/jospt.2008.0302. 18434670

[4] Irving, Damien B. , Jill L. Cook , Mark A. Young and Hylton B. Menz . 2008. “Impact of Chronic Plantar Heel Pain on Health‐Related Quality of Life.” Journal of the American Podiatric Medical Association 98(4): 283–289. 10.7547/0980283. 18685048

[5] Cotchett, Matthew , Michael Skovdal Rathleff , Matthew Dilnot , Karl B. Landorf , Dylan Morrissey and Christian Barton . 2020. “Lived Experience and Attitudes of People with Plantar Heel Pain: a Qualitative Exploration.” Journal of Foot and Ankle Research 13: 1–9. 10.1186/s13047-020-0377-3. 32143679 PMC7059663

[6] Gulle, Halime , Dylan Morrissey , Xiang Li Tan , Matthew Cotchett , Stuart Charles Miller , Aleksandra Birn Jeffrey and Trevor Prior . 2023. “Predicting the Outcome of Plantar Heel Pain in Adults: A Systematic Review of Prognostic Factors.” Journal of Foot and Ankle Research 16(1): 28. 10.1186/s13047-023-00626-y. 37173686 PMC10176769

[7] Morrissey, Dylan , Matthew Cotchett , Ahmed Said J'Bari , Trevor Prior , Ian B. Griffiths , Michael Skovdal Rathleff , Halime Gulle , Bill Vicenzino and Christian J. Barton . 2021. “Management of Plantar Heel Pain: a Best Practice Guide Informed by a Systematic Review, Expert Clinical Reasoning and Patient Values.” British Journal of Sports Medicine 55(19): 1106–1118: bjsports‐2019‐101970. 10.1136/bjsports-2019-101970. 33785535 PMC8458083

[8] Babatunde, Opeyemi O. , Amardeep Legha , Chris Littlewood , Linda S. Chesterton , Martin J. Thomas , Hylton B. Menz , Danielle van der Windt and Edward Roddy . 2019. “Comparative Effectiveness of Treatment Options for Plantar Heel Pain: a Systematic Review with Network Meta‐Analysis.” British Journal of Sports Medicine 53(3): 182–194. 10.1136/bjsports-2017-098998. 29954828

[9] Salvioli, Stefano , Maddalena Guidi and Giulia Marcotulli . 2017. “The Effectiveness of Conservative, Non‐pharmacological Treatment, of Plantar Heel Pain: a Systematic Review with Meta‐Analysis.” The Foot 33: 57–67. 10.1016/j.foot.2017.05.004. 29126045

[10] Hansen, Liselotte , Thøger Persson Krogh , Torkell Ellingsen , Lars Bolvig and Ulrich Fredberg . 2018. “Long‐term Prognosis of Plantar Fasciitis: a 5‐to 15‐year Follow‐Up Study of 174 Patients with Ultrasound Examination.” Orthopaedic Journal of Sports Medicine 6(3): 2325967118757983. 10.1177/2325967118757983. 29536022 PMC5844527

[11] Vertuccio, A. , D. Perugia , Rm Lanzetti , A. Massimiani , A. Lacopo , Sm Nusca , R. Baldini , et al. 2021. “Demographic and Clinical Factors Predict Focused Shockwave Therapy Results on Patients with Plantar Fasciitis. A Multilevel Analysis.” Muscles, Ligaments & Tendons Journal (MLTJ) 11(3): 376. 10.32098/mltj.03.2021.02.

[12] Fleischer, Adam E. , Rachel H. Albright , Ryan T. Crews , Tatiana Kelil and James S. Wrobel . 2015. “Prognostic Value of Diagnostic Sonography in Patients with Plantar Fasciitis.” Journal of Ultrasound in Medicine 34(10): 1729–1735. 10.7863/ultra.15.14.10062. 26307122

[13] McClinton, Shane M. , Joshua A. Cleland and Timothy W. Flynn . 2015. “Predictors of Response to Physical Therapy Intervention for Plantar Heel Pain.” Foot & Ankle International 36(4): 408–416. 10.1177/1071100714558508. 25367253

[14] Creighton, Douglas S. and Varick L. Olson . 1987. “Evaluation of Range of Motion of the First Metatarsophalangeal Joint in Runners with Plantar Faciitis.” Journal of Orthopaedic & Sports Physical Therapy 8(7): 357–361. 10.2519/jospt.1987.8.7.357. 18797043

[15] Wearing, Scott C. , James E. Smeathers , Bede Yates , Patrick M. Sullivan , Stephen R. Urry and Philip Dubois . 2004. “Sagittal Movement of the Medial Longitudinal Arch Is Unchanged in Plantar Fasciitis.” Medicine & Science in Sports & Exercise 36(10): 1761–1767. 10.1249/01.mss.0000142297.10881.11. 15595298

[16] Ozdemir, Huseyin , Erhan Yilmaz , Ayse Murat , Lokman Karakurt , A. Kursad Poyraz and Erkin Ogur . 2005. “Sonographic Evaluation of Plantar Fasciitis and Relation to Body Mass Index.” European Journal of radiology 54(3): 443–447. 10.1016/j.ejrad.2004.09.004. 15899349

[17] Kibler, W. Ben , Cindy Goldberg and T. Jeff Chandler . 1991. “Functional Biomechanical Deficits in Running Athletes with Plantar Fasciitis.” The American Journal of Sports Medicine 19(1): 66–71. 10.1177/036354659101900111. 1672577

[18] Sobel, David S. 1995. “Rethinking Medicine: Improving Health Outcomes with Cost‐Effective Psychosocial Interventions.” Psychosomatic Medicine 57(3): 234–244. 10.1097/00006842-199505000-00005. 7652124

[19] Suvinen, Tuija I. , Peter C. Reade , Pentti Kemppainen , Mauno Könönen and Samuel F. Dworkin . 2005. “Review of Aetiological Concepts of Temporomandibular Pain Disorders: towards a Biopsychosocial Model for Integration of Physical Disorder Factors with Psychological and Psychosocial Illness Impact Factors.” European Journal of Pain 9(6): 613–633. 10.1016/j.ejpain.2005.01.012. 15978854

[20] Hansen, Liselotte , Thøger Persson Krogh , Torkell Ellingsen , Lars Bolvig and Ulrich Fredberg . 2018. “Long‐term Prognosis of Plantar Fasciitis: a 5‐to 15‐year Follow‐Up Study of 174 Patients with Ultrasound Examination.” Orthopaedic Journal of Sports Medicine 6(3): 2325967118757983. 10.1177/2325967118757983. 29536022 PMC5844527

[21] Bumann, A. , W. Banzer and J. Fleckenstein . 2020. “Prevalence of Biopsychosocial Factors of Pain in 865 Sports Students of the Dach (Germany, Austria, Switzerland) Region–A Cross‐Sectional Survey.” Journal of Sports Science and Medicine 19(2): 323. 32390726 PMC7196754

[22] Caneiro, J. , P. B. O'Sullivan , E. M. Roos , A. J. Smith , P. Choong , M. Dowsey , D. J. Hunter , J. Kemp , J. Rodriguez and S. Lohmander . 2020. Three Steps to Changing the Narrative about Knee Osteoarthritis Care: A Call to Action. BMJ Publishing Group Ltd and British Association of Sport and Exercise Medicine. 10.1136/bjsports-2019-101328 31484634

[23] Mitchell, Tim , Peter B. O'Sullivan , Anne Smith , Angus F. Burnett , Leon Straker , Jenny Thornton and Cobie J. Rudd . 2009. “Biopsychosocial Factors Are Associated with Low Back Pain in Female Nursing Students: a Cross‐Sectional Study.” International Journal of Nursing Studies 46(5): 678–688. 10.1016/j.ijnurstu.2008.11.004. 19118828

[24] Kamper, Steven J. , Andreas T. Apeldoorn , Alessandro Chiarotto , Rob J. E. M. Smeets , Raymond W. J. G. Ostelo , Jaime Guzman and Maurits W. van Tulder . 2014. “Multidisciplinary Biopsychosocial Rehabilitation for Chronic Low Back Pain.” Cochrane Database of Systematic Reviews 2014(9). 10.1002/14651858.cd000963.pub3. PMC10945502 25180773

[25] van Leeuwen, K. D. B. , J. Rogers , T. Winzenberg and M. van Middelkoop . 2016. “Higher Body Mass Index Is Associated with Plantar Fasciopathy/‘plantar Fasciitis’: Systematic Review and Meta‐Analysis of Various Clinical and Imaging Risk Factors.” British Journal of Sports Medicine 50(16): 972–981. 10.1136/bjsports-2015-094695. 26644427

[26] Irving, D. , J. Cook and H. Menz . 2006. “Factors Associated with Chronic Plantar Heel Pain: A Matched Case–Control Study.” Journal of Science and Medicine in Sport 9: 7. 10.1016/j.jsams.2006.12.013. 16584917

[27] Cotchett, Matthew P. , Glen Whittaker and Bircan Erbas . 2015. “Psychological Variables Associated with Foot Function and Foot Pain in Patients with Plantar Heel Pain.” Clinical Rheumatology 34(5): 957–964. 10.1007/s10067-014-2565-7. 24647980

[28] Cotchett, Matthew , Shannon E. Munteanu and Karl B. Landorf . 2016. “Depression, Anxiety and Stress in People with and without Plantar Heel Pain.” Foot & Ankle International 37(8): 816–821. 10.1177/1071100716646630. 27137796

[29] Cotchett, Matthew , Angus Lennecke , Virginia G. Medica , Glen A. Whittaker and Daniel R. Bonanno . 2017. “The Association between Pain Catastrophising and Kinesiophobia with Pain and Function in People with Plantar Heel Pain.” The Foot 32: 8–14. 10.1016/j.foot.2017.03.003. 28605621

[30] Tayfur, Abdulhamit , Ateş Şendil , Atilla Çağatay Sezik , J.‐François Kaux , Igor Sancho , Guillaume Le Sant , Gürhan Dönmez , et al. 2023. “Self‐reported Bio‐Psycho‐Social Factors Partially Distinguish Patellar Tendinopathy from Other Knee Problems and Explain Patellar Tendinopathy Severity in Jumping Athletes: A Case‐Control Study.” Physical Therapy in Sport 61: 57–65. 10.1016/j.ptsp.2023.02.009. 36898283

[31] Delen, Mehmet , Ateş Şendil , J.‐François Kaux , Carles Pedret , Guillaume Le Sant , Jessica Pawson , Stuart Charles Miller , Aleksandra Birn‐Jeffery and Dylan Morrissey . 2023. “Self‐reported Bio‐psycho‐social Factors Partially Distinguish Rotator Cuff Tendinopathy from Other Shoulder Problems and Explain Shoulder Severity: A Case‐control Study.” Musculoskeletal Care 21(1): 175–188. 10.1002/msc.1679. 35983898

[32] Gulle, Halime , Trevor Prior , Stuart Miller , Aleksandra V. Birn‐Jeffery and Dylan Morrissey . 2021. “Online Questionnaire, Clinical and Biomechanical Measurements for Outcome Prediction of Plantar Heel Pain: Feasibility for a Cohort Study.” Journal of Foot and Ankle Research 14(1): 1–13. 10.1186/s13047-021-00472-w. 33902655 PMC8077700

[33] Boudreau, Shellie A. , Susanne Badsberg , Steffan W. Christensen and Line L. Egsgaard . 2016. “Digital Pain Drawings.” The Clinical Journal of Pain 32(2): 139–145. 10.1097/ajp.0000000000000230. 25756558

[34] Boudreau, S. A. , R. Spence , G. Vasov and L. L. Egsgaard . 2014. “Feature Extraction APP for Pain Profiles.” In Replace, Repair, Restore, Relieve–Bridging Clinical and Engineering Solutions in Neurorehabilitation, 853–854. Springer.

[35] Martin, Robroy L. and James J. Irrgang . 2007. “A Survey of Self‐Reported Outcome Instruments for the Foot and Ankle.” Journal of Orthopaedic & Sports Physical Therapy 37(2): 72–84. 10.2519/jospt.2007.2403. 17366962

[36] Bennett, P. J. , C. Patterson , S. Wearing and T. Baglioni . 1998. “Development and Validation of a Questionnaire Designed to Measure Foot‐Health Status.” Journal of the American Podiatric Medical Association 88(9): 419–428. 10.7547/87507315-88-9-419. 9770933

[37] Riskowski, Jody L. , Thomas J. Hagedorn and Marian T. Hannan . 2011. “Measures of Foot Function, Foot Health and Foot Pain: American Academy of Orthopedic Surgeons Lower Limb Outcomes Assessment: Foot and Ankle Module (AAOS‐FAM), Bristol Foot Score (BFS), Revised Foot Function Index (FFI‐R), Foot Health Status Questionnaire (FHSQ), Manchester Foot Pain and Disability Index (MFPDI), Podiatric Health Questionnaire (PHQ) and Rowan Foot Pain Assessment (ROFPAQ).” Arthritis Care & Research 63(S11): S229–S239. 10.1002/acr.20554. 22588747 PMC4155931

[38] Dolan, Paul . 1997. “Modeling Valuations for EuroQol Health States.” Medical Care 35(11): 1095–1108. 10.1097/00005650-199711000-00002. 9366889

[39] Sullivan, Michael J. L. , Scott R. Bishop and Jayne Pivik . 1995. “The Pain Catastrophizing Scale: Development and Validation.” Psychological Assessment 7(4): 524–532. 10.1037//1040-3590.7.4.524.

[40] Scott, Whitney , Timothy H. Wideman and Michael J. L. Sullivan . 2014. “Clinically Meaningful Scores on Pain Catastrophizing before and after Multidisciplinary Rehabilitation: a Prospective Study of Individuals with Subacute Pain after Whiplash Injury.” The Clinical Journal of Pain 30(3): 183–190. 10.1097/ajp.0b013e31828eee6c. 23552561

[41] Osman, Augustine , Francisco X. Barrios , Beverly A. Kopper , Wendy Hauptmann , Jewel Jones and Elizabeth O'Neill . 1997. “Factor Structure, Reliability and Validity of the Pain Catastrophizing Scale.” Journal of Behavioral Medicine 20(6): 589–605. 10.1023/a:1025570508954. 9429990

[42] Mccracken, Lance M. , Richard T. Gross , James Aikens and Clm Carnrike, Jr . 1996. “The Assessment of Anxiety and Fear in Persons with Chronic Pain: a Comparison of Instruments.” Behaviour Research and Therapy 34(11–12): 927–933. 10.1016/s0005-7967(96)00057-5. 8990544

[43] Jacob, Tamar , Mario Baras , Aviva Zeev and Leon Epstein . 2001. “Low Back Pain: Reliability of a Set of Pain Measurement Tools.” Archives of Physical Medicine and Rehabilitation 82(6): 735–742. 10.1053/apmr.2001.22623. 11387576

[44] Swinkels‐Meewisse, Ejcm , Rahm Swinkels , Alm Verbeek , Jws Vlaeyen and Rab Oostendorp . 2003. “Psychometric Properties of the Tampa Scale for Kinesiophobia and the Fear‐Avoidance Beliefs Questionnaire in Acute Low Back Pain.” Manual Therapy 8(1): 29–36. 10.1054/math.2002.0484. 12586559

[45] Cleland, Joshua A. , Julie M. Fritz and Gerard P. Brennan . 2008. “Predictive Validity of Initial Fear Avoidance Beliefs in Patients with Low Back Pain Receiving Physical Therapy: Is the FABQ a Useful Screening Tool for Identifying Patients at Risk for a Poor Recovery?” European Spine Journal 17(1): 70–79. 10.1007/s00586-007-0511-y. 17926072 PMC2365529

[46] Neblett, Randy , Howard Cohen , YunHee Choi , Meredith M. Hartzell , Mark Williams , Tom G. Mayer and Robert J. Gatchel . 2013. “The Central Sensitization Inventory (CSI): Establishing Clinically Significant Values for Identifying Central Sensitivity Syndromes in an Outpatient Chronic Pain Sample.” The Journal of Pain 14(5): 438–445. 10.1016/j.jpain.2012.11.012. 23490634 PMC3644381

[47] Crespo, Carlos J. , Ellen Smit , Ross E. Andersen , Olivia Carter‐Pokras and Barbara E. Ainsworth . 2000. “Race/ethnicity, Social Class and Their Relation to Physical Inactivity during Leisure Time: Results from the Third National Health and Nutrition Examination Survey, 1988–1994.” American Journal of Preventive Medicine 18(1): 46–53. 10.1016/s0749-3797(99)00105-1. 10808982

[48] Nguyen, Jennifer , Michael Moorhouse , Barbara Curbow , Juliette Christie , Kim Walsh‐Childers and Sabrina Islam . 2016. “Construct Validity of the eHealth Literacy Scale (eHEALS) Among Two Adult Populations: a Rasch Analysis.” JMIR Public Health and Surveillance 2(1): e24. 10.2196/publichealth.4967. 27244771 PMC4909391

[49] Herrmann, Stephen D. , Kristin J. Heumann , Cheryl A. Der Ananian and Barbara E. Ainsworth . 2013. “Validity and Reliability of the Global Physical Activity Questionnaire (GPAQ).” Measurement in Physical Education and Exercise Science 17(3): 221–235. 10.1080/1091367x.2013.805139.

[50] Sullivan, Gail M. and Richard Feinn . 2012. “Using Effect Size—Or Why the P Value Is Not Enough.” Journal of Graduate Medical Education 4(3): 279–282. 10.4300/jgme-d-12-00156.1. 23997866 PMC3444174

[51] O’brien, Robert M. 2007. “A Caution Regarding Rules of Thumb for Variance Inflation Factors.” Quality and Quantity 41(5): 673–690. 10.1007/s11135-006-9018-6.

[52] Turk, Dennis C. , Robert H. Dworkin , Dennis Revicki , Gale Harding , Laurie B. Burke , David Cella , Charles S. Cleeland , et al. 2008. “Identifying Important Outcome Domains for Chronic Pain Clinical Trials: an IMMPACT Survey of People with Pain.” PAIN® 137(2): 276–285. 10.1016/j.pain.2007.09.002. 17937976

[53] Katz, Nathaniel . 2002. “The Impact of Pain Management on Quality of Life.” Journal of Pain and Symptom Management 24(1): S38–S47. 10.1016/s0885-3924(02)00411-6. 12204486

[54] Fernández‐Lao, Carolina , Noelia Galiano‐Castillo , Irene Cantarero‐Villanueva , Lydia Martín‐Martín , Nicolás Prados‐Olleta and Manuel Arroyo‐Morales . 2016. “Analysis of Pressure Pain Hypersensitivity, Ultrasound Image and Quality of Life in Patients with Chronic Plantar Pain: a Preliminary Study.” Pain Medicine 17(8): 1530–1541. 10.1093/pm/pnv022. 26814301

[55] Plaza‐Manzano, Gustavo , Marta Ríos‐León , Patricia Martín‐Casas , Lars Arendt‐Nielsen , César Fernández‐de‐las‐Peñas and Ricardo Ortega‐Santiago . 2019. “Widespread Pressure Pain Hypersensitivity in Musculoskeletal and Nerve Trunk Areas as a Sign of Altered Nociceptive Processing in Unilateral Plantar Heel Pain.” The Journal of Pain 20(1): 60–67. 10.1016/j.jpain.2018.08.001. 30121357

[56] Wheeler, Patrick C. 2019. “Up to a Quarter of Patients with Certain Chronic Recalcitrant Tendinopathies May Have Central Sensitisation: a Prospective Cohort of More Than 300 Patients.” British Journal of Pain 13(3): 137–144. 10.1177/2049463718800352. 31308939 PMC6613072

[57] Kamaleri, Yusman , Bård Natvig , Camilla M. Ihlebaek , Jurate Saltyte Benth and Dag Bruusgaard . 2009. “Change in the Number of Musculoskeletal Pain Sites: a 14‐year Prospective Study.” PAIN® 141(1–2): 25–30. 10.1016/j.pain.2008.09.013. 18977088

[58] Werner, Robert A. , Nancy Gell , Anne Hartigan , Neal Wiggerman and William M. Keyserling . 2010. “Risk Factors for Plantar Fasciitis Among Assembly Plant Workers.” PM&R 2(2): 110–116. 10.1016/j.pmrj.2009.11.012. 20193937

[59] Wijnhoven, Hanneke A. H. , Henrica C. W. de Vet and Susan J. H. Picavet . 2006. “Explaining Sex Differences in Chronic Musculoskeletal Pain in a General Population.” Pain 124(1–2): 158–166. 10.1016/j.pain.2006.04.012. 16716517

[60] Bartley, E. J. and R. B. Fillingim . 2013. “Sex Differences in Pain: a Brief Review of Clinical and Experimental Findings.” British Journal of Anaesthesia 111(1): 52–58. 10.1093/bja/aet127. 23794645 PMC3690315

[61] Kuyucu, Ersin , Figen Koçyiğit and Mehmet Erdil . 2015. “The Association of Calcaneal Spur Length and Clinical and Functional Parameters in Plantar Fasciitis.” International Journal of Surgery 21: 28–31. 10.1016/j.ijsu.2015.06.078. 26184993

[62] Ong, W.‐Yi , Christian S. Stohler and Deron R. Herr . 2019. “Role of the Prefrontal Cortex in Pain Processing.” Molecular Neurobiology 56(2): 1137–1166. 10.1007/s12035-018-1130-9. 29876878 PMC6400876

[63] Bruehl, Stephen and Ok Yung Chung . 2006. “Psychological and Behavioral Aspects of Complex Regional Pain Syndrome Management.” The Clinical Journal of Pain 22(5): 430–437. 10.1097/01.ajp.0000194282.82002.79. 16772797

[64] Klein, Sandra E. , Ann Marie Dale , Marcie Harris Hayes , Jeffrey E. Johnson , Jeremy J. McCormick and Brad A. Racette . 2012. “Clinical Presentation and Self‐Reported Patterns of Pain and Function in Patients with Plantar Heel Pain.” Foot & Ankle International 33(9): 693–698. 10.3113/fai.2012.0693. 22995253 PMC4083061

[65] Aldridge, T. 2004. “Diagnosing Heel Pain in Adults.” American Family Physician 70(2): 332–338. 15291091

[66] Yi, Tae Im , Ga Eun Lee , In Seok Seo , Won Seok Huh , Tae Hee Yoon and Bo Ra Kim . 2011. “Clinical Characteristics of the Causes of Plantar Heel Pain.” Annals of Rehabilitation Medicine 35(4): 507. 10.5535/arm.2011.35.4.507. 22506166 PMC3309235

[67] Rogers, Jason , Graeme Jones , Jill L. Cook , Karen Wills , Aroub Lahham and Tania M. Winzenberg . 2021. “Chronic Plantar Heel Pain Is Principally Associated with Waist Girth (Systemic) and Pain (Central) Factors, Not Foot Factors: A Case‐Control Study.” Journal of Orthopaedic & Sports Physical Therapy 51(9): 449–458. 10.2519/jospt.2021.10018. 33962520

[68] Thomas, James L. , Jeffrey C. Christensen , Steven R. Kravitz , Robert W. Mendicino , John M. Schuberth , John V. Vanore , Lowell Scott Weil, Sr , Howard J. Zlotoff , Richard Bouché and Jeffrey Baker . 2010. “The Diagnosis and Treatment of Heel Pain: a Clinical Practice Guideline–Revision 2010.” Journal of Foot & Ankle Surgery 49(3): S1–S19. 10.1053/j.jfas.2010.01.001. 20439021

[69] Rio, E. , S. Mayes and J. Cook . 2015. “Heel Pain: a Practical Approach.” Australian Family Physician 44(3): 96–101. 25770572

[70] Lui, Eric . 2010. “Systemic Causes of Heel Pain.” Clinics in Podiatric Medicine and Surgery 27(3): 431–441. 10.1016/j.cpm.2010.04.004. 20691375

[71] Zale, Emily L. , Krista L. Lange , Sherecce A. Fields and Joseph W. Ditre . 2013. “The Relation between Pain‐Related Fear and Disability: a Meta‐Analysis.” The Journal of Pain 14(10): 1019–1030. 10.1016/j.jpain.2013.05.005. 23850095 PMC3791167

[72] Wideman, Timothy H. and Michael J. L. Sullivan . 2011. “Differential Predictors of the Long‐Term Levels of Pain Intensity, Work Disability, Healthcare Use and Medication Use in a Sample of Workers’ Compensation Claimants.” PAIN® 152(2): 376–383. 10.1016/j.pain.2010.10.044. 21147513

[73] Domenech, Julio , Vicente Sanchis‐Alfonso , Laura López and Begoña Espejo . 2013. “Influence of Kinesiophobia and Catastrophizing on Pain and Disability in Anterior Knee Pain Patients.” Knee Surgery, Sports Traumatology, Arthroscopy 21(7): 1562–1568. 10.1007/s00167-012-2238-5. 23081711

[74] Irving, Damien B. , Jill L. Cook , Mark A. Young and Hylton B. Menz . 2007. “Obesity and Pronated Foot Type May Increase the Risk of Chronic Plantar Heel Pain: a Matched Case‐Control Study.” BMC Musculoskeletal Disorders 8(1): 1–8. 10.1186/1471-2474-8-41. 17506905 PMC1884155

[75] McMillan, Andrew , Karl Landorf , Joanna Barrett , Hylton Menz and Adam Bird . 2011. “Diagnostic Imaging for Chronic Plantar Heel Pain: a Systematic Review and Meta‐Analysis.” Journal of Foot and Ankle Research 4(1): 1. 10.1186/1757-1146-4-s1-p40. 19912628 PMC2784446

[76] McMillan, A. M. , K. B. Landorf , M. F. Gilheany , A. R. Bird , A. D. Morrow and H. B. Menz . 2012. “Ultrasound Guided Corticosteroid Injection for Plantar Fasciitis: Randomised Controlled Trial.” BMJ 344(may22 1): e3260. 10.1136/bmj.e3260. 22619193

[77] Radford, Joel A. , Karl B. Landorf , Rachelle Buchbinder and Catherine Cook . 2007. “Effectiveness of Calf Muscle Stretching for the Short‐Term Treatment of Plantar Heel Pain: a Randomised Trial.” BMC Musculoskeletal Disorders 8(1): 1–8. 10.1186/1471-2474-8-36. 17442119 PMC1867816

[78] Landorf, Karl B. , A.‐Maree Keenan and Robert D. Herbert . 2006. “Effectiveness of Foot Orthoses to Treat Plantar Fasciitis: a Randomized Trial.” Archives of Internal Medicine 166(12): 1305–1310. 10.1001/archinte.166.12.1305. 16801514

