# Supplementary material for: The association of demographic, psychological, social and activity factors with foot health in people with plantar heel pain
Source: J Foot Ankle Res. 2024 Dec 11;17(4):e70022. doi: 10.1002/jfa2.70022 (PMC11634547; doi:10.1002/jfa2.70022)
Supplement: Supplementary file 1 — Supplementary Material [file JFA2-17-e70022-s001.docx]

# **SUPPLEMENTARY FILE**

## **1. STROBE checklist**

Supplementary table 1: STROBE Statement—checklist of items that should be included in reports of observational studies

|  | Item No | Recommendation | Page no | Relevant text from manuscript |
| --- | --- | --- | --- | --- |
| **Title and abstract** | 1 | (*a*) Indicate the study’s design with a commonly used term in the title or the abstract | 1 | A cross-sectional study |
|  |  | (*b*) Provide in the abstract an informative and balanced summary of what was done and what was found | 1 | We collected data from 235 participants, including 136 people with PHP... (Methods in abstract). The strongest factor associated with foot health severity was overall… (Results in abstract). |
| Introduction | | |  |  |
| Background/rationale | 2 | Explain the scientific background and rationale for the investigation being reported | 2 | … there is no research that has specifically evaluated the wide range of plausible biopsychosocial factors that is required to inform more nuanced intervention development. |
| Objectives | 3 | State specific objectives, including any prespecified hypotheses | 2 | The objectives were to better understand the severity of compromised foot health in this population |
| Methods | | |  |  |
| Study design | 4 | Present key elements of study design early in the paper | 3-7 | Materials and methods section |
| Setting | 5 | Describe the setting, locations, and relevant dates, including periods of recruitment, exposure, follow-up, and data collection | 3 | The sample included 235 people who were recruited via advertising in hospitals and physiotherapy clinics, posters in public areas, and social media outlets over a year in 2019/20. |
| Participants | 6 | (*a*) *Cohort study*—Give the eligibility criteria, and the sources and methods of selection of participants. Describe methods of follow-up  *Case-control study*—Give the eligibility criteria, and the sources and methods of case ascertainment and control selection. Give the rationale for the choice of cases and controls  *Cross-sectional study*—Give the eligibility criteria, and the sources and methods of selection of participants | 3-4 | The inclusion criteria were having a clinical diagnosis of PHP or another clinically diagnosed ankle or foot musculoskeletal condition within the last 6 months. A podiatrist with over 30 years’ clinic experience (TP) and a clinician’s (DC) team diagnosed 72 percent of both groups of conditions based on reported symptoms and clinical examination. Subjects…. |
|  |  | (*b*) *Cohort study*—For matched studies, give matching criteria and number of exposed and unexposed  *Case-control study*—For matched studies, give matching criteria and the number of controls per case |  | Not applicable |
| Variables | 7 | Clearly define all outcomes, exposures, predictors, potential confounders, and effect modifiers. Give diagnostic criteria, if applicable | 4 | Methods- Measures section |
| Data sources/ measurement | 8* | For each variable of interest, give sources of data and details of methods of assessment (measurement). Describe comparability of assessment methods if there is more than one group | 4 | Methods- Main patient reported outcome measure, Quality of Life, Biomedical characteristics, psychological measures, Social related measures and activity related measures. |
|  | 9 | Describe any efforts to address potential sources of bias | 3 | Methods- Participants & screening process |
| Study size | 10 | Explain how the study size was arrived at | 7 | The sample size was based on the ratio of the number of individuals with the outcome event to the number of candidate predictors, referred to as the events per variable (EPV). An EPV of 20 was determined for multivariable linear and logistic regression analyses according to published research |
| Quantitative variables | 11 | Explain how quantitative variables were handled in the analyses. If applicable, describe which groupings were chosen and why | 6-7 | Methods- Data Analysis & Statistical analyses |
| Statistical methods | 12 | (*a*) Describe all statistical methods, including those used to control for confounding | 7 | Methods- Statistical analyses |
|  |  | (*b*) Describe any methods used to examine subgroups and interactions | 7 | Methods- Statistical analyses |
|  |  | (*c*) Explain how missing data were addressed |  | Not applicable |
|  |  | (*d*) *Cohort study*—If applicable, explain how loss to follow-up was addressed  *Case-control study*—If applicable, explain how matching of cases and controls was addressed  *Cross-sectional study*—If applicable, describe analytical methods taking account of sampling strategy | 7 | Methods- Statistical analyses |
|  |  | (*e*) Describe any sensitivity analyses | 7 | Model fit was tested with Hosmer-Lemeshow. Accuracy, specificity and sensitivity of the model were also assessed |
| Results | | |  |  |
| Participants | 13* | (a) Report numbers of individuals at each stage of study—eg numbers potentially eligible, examined for eligibility, confirmed eligible, included in the study, completing follow-up, and analysed | 7-8 | Results- sample characteristics section |
|  |  | (b) Give reasons for non-participation at each stage |  | Methods- Figure 1 |
|  |  | (c) Consider use of a flow diagram |  | Methods- Figure 1 |
| Descriptive data | 14* | (a) Give characteristics of study participants (eg demographic, clinical, social) and information on exposures and potential confounders | 7-8 | Results- sample characteristics & table 1 |
|  |  | (b) Indicate number of participants with missing data for each variable of interest |  | Not applicable |
|  |  | (c) *Cohort study*—Summarise follow-up time (eg, average and total amount) |  | Not applicable |
| Outcome data | 15* | *Cohort study*—Report numbers of outcome events or summary measures over time |  | Not applicable |
|  |  | *Case-control study—*Report numbers in each exposure category, or summary measures of exposure | 7-8 | Results- sample characteristics section |
|  |  | *Cross-sectional study—*Report numbers of outcome events or summary measures |  |  |
| Main results | 16 | (*a*) Give unadjusted estimates and, if applicable, confounder-adjusted estimates and their precision (eg, 95% confidence interval). Make clear which confounders were adjusted for and why they were included | 10 | Results- Multivariable Linear regression for severity of PHP & Multivariable Logistic regression comparing people with PHP and OFP & supplement file |
|  |  | (*b*) Report category boundaries when continuous variables were categorized |  | Not applicable |
|  |  | (*c*) If relevant, consider translating estimates of relative risk into absolute risk for a meaningful time period |  | Not applicable |
| Other analyses | 17 | Report other analyses done—eg analyses of subgroups and interactions, and sensitivity analyses | 11 | Model fit was good (Hosmer-Lemeshow test= 0.75, p<0.001) with acceptable accuracy (AUC=0.78), specificity (69.8%) and sensitivity (70.1%)… |
| Discussion | | |  |  |
| Key results | 18 | Summarise key results with reference to study objectives | 11 | Discussion- Key results (first paragraph of discussion) |
| Limitations | 19 | Discuss limitations of the study, taking into account sources of potential bias or imprecision. Discuss both direction and magnitude of any potential bias | 14 | Discussion- Limitation |
| Interpretation | 20 | Give a cautious overall interpretation of results considering objectives, limitations, multiplicity of analyses, results from similar studies, and other relevant evidence | 13-14 | Discussion- Severity of plantar heel pain & Comparison between people with PHP and OFP sections |
| Generalisability | 21 | Discuss the generalisability (external validity) of the study results | 13 | Discussion- Strengths, limitations and future Directions |
| Other information | | |  |  |
| Funding | 22 | Give the source of funding and the role of the funders for the present study and, if applicable, for the original study on which the present article is based |  | Entered during submission process |

## **2. Correlation matrix**

Supplementary table 2: Correlation matrix between independent variables

| **Correlations between explanatory factors** | | | | | | | | | | | | | |
| --- | --- | --- | --- | --- | --- | --- | --- | --- | --- | --- | --- | --- | --- |
| VARIABLES | (1) | (2) | (3) | (4) | (5) | (6) | (7) | (8) | (9) | (10) | (11) | (12) | (13) |
| (1) Age | ̶ |  |  |  |  |  |  |  |  |  |  |  |  |
| (2) BMI | 0.1055 | ̶ |  |  |  |  |  |  |  |  |  |  |  |
| (3) Catastrophization | 0.0488 | 0.0916 | ̶ |  |  |  |  |  |  |  |  |  |  |
| (4) Sensitization | 0.0716 | 0.1951 | 0.4934 | ̶ |  |  |  |  |  |  |  |  |  |
| (5) Fear avoidance-work | -0.0039 | 0.0615 | 0.4200 | 0.2972 | ̶ |  |  |  |  |  |  |  |  |
| (6) Fear avoidance-PA | 0.0591 | 0.0886 | 0.4965 | 0.2944 | 0.1429 | ̶ |  |  |  |  |  |  |  |
| (7) EQ5D5L-index | -0.0982 | -0.1543 | -0.5579 | -0.5028 | -0.2892 | -0.2740 | ̶ |  |  |  |  |  |  |
| (8) Pain Duration | 0.0184 | -0.0648 | 0.1741 | 0.1009 | 0.1190 | 0.0717 | -0.1298 | ̶ |  |  |  |  |  |
| (9) Pain Severity | 0.0233 | 0.2242 | 0.3965 | 0.1507 | 0.2220 | 0.1129 | -0.2995 | 0.1147 | ̶ |  |  |  |  |
| (10) Num. of Comorbidity | 0.1584 | -0.0261 | 0.3201 | 0.4047 | 0.0914 | 0.1556 | -0.3091 | 0.0703 | 0.1362 | ̶ |  |  |  |
| (11) Hours Standing | -0.0405 | -0.0446 | -0.0291 | 0.0326 | 0.0948 | -0.0382 | 0.0891 | -0.0447 | 0.0449 | 0.0295 | ̶ |  |  |
| (12) Activity Level | -0.1687 | -0.2437 | 0.0620 | -0.0406 | 0.2050 | 0.0884 | 0.0074 | 0.1742 | 0.1737 | -0.0329 | 0.3401 | ̶ |  |
| (13) Health literacy | -0.1182 | 0.0022 | -0.0133 | -0.0179 | -0.0429 | -0.1061 | 0.0461 | 0.0665 | -0.0988 | -0.0003 | -0.0783 | 0.0947 | ̶ |
| (14) Footwear | -0.0597 | 0.1192 | 0.0592 | 0.1968 | 0.0638 | 0.0615 | -0.1726 | -0.0389 | 0.0633 | 0.0865 | 0.0765 | 0.0113 | -0.0449 |

## **3. Univariate analyses results for linear regression**

Supplementary table 3: Univariate analyses results for linear regression (n=136, people with PHP) R^2^: statistical measure that represents the proportion of the variance for a dependent variable that's explained by an independent variable or variables in a regression model. The dependent variable is general foot health subscale of FHSQ, which is 0-100 scale, indicating worse to better foot health score. Negative coefficients mean an increased severity of PHP condition, while positive coefficients mean a decreased severity of PHP condition. Key: mins= minutes, CI: Confidence Interval, β=Beta, Coef= coefficient. Bold variables are indicating statistically significant results. ‡Handled as continuous in the models using the combined categories, assuming linearity and the coefficients are per category increase.

| Potential Predictors | R^2^ | Beta-coefficient | Std. Error | P > I t I |
| --- | --- | --- | --- | --- |
| Quality of Life | **0.15**  **15** | **0.38** | **8.54** | **<0.000** |
| Demographics |  |  |  |  |
| Age | 0.01 | -.12 | 0.16 | 0.15 |
| BMI | 0.01 | -.11 | 0.49 | 0.17 |
| Sex (ref: male) | **0.03** | **-.20** | **4.51** | **0.01** |
| Ethnicity (ref others and PNTS) | 0.008 |  |  |  |
| White |  | .09 | 5.06 | 0.31 |
| Asian |  | -.01 | 6.93 | 0.89 |
| Biomedical |  |  |  |  |
| Morning Pain duration, mins. | **0.02** | **-.16** | **0.03** | **0.05** |
| Morning Pain Severity, VAS | **0.09** | **-.30** | **0.06** | **<0.000** |
| Disease duration^‡^ | **0.04** | **-.20** | **1.01** | **0.01** |
| Onset of Pain (Sudden: Gradual: Other) | 0.008 | .09 | 4.69 | 0.27 |
| Comorbidity (ref: none) | **0.07** |  |  |  |
| Musculoskeletal d. |  | **-.25** | **6.65** | **0.005** |
| Systemic d. |  | .04 | 5.39 | 0.64 |
| Psychological d. |  | -.10 | 5.70 | 0.24 |
| Number of Comorbidity | 0.003 | -.06 | 2.81 | 0.47 |
| Back pain presence, (ref: no) | **0.06** |  |  |  |
| Yes (Current, recurrent) |  | **-.31** | **5.45** | **0.004** |
| Yes (Previously) |  | **-.20** | **6.06** | **0.05** |
| Pain in walking (ref: no change) | 0.004 |  |  |  |
| Worse |  | -.11 | 8.24 | 0.48 |
| Better |  | -.06 | 8.54 | 0.66 |
| Pain in standing (ref: no change) | 0.01 |  |  |  |
| Worse |  | -.06 | 5.59 | 0.52 |
| Better |  | .06 | 10.84 | 0.47 |
| Pain in sitting (ref: no change) | 0.03 |  |  |  |
| Worse |  | **-.23** | **6.08** | **0.03** |
| Better |  | -.07 | 5.49 | 0.48 |
| Number of investigations | 0.01 | -.12 | 2.21 | 0.16 |
| Number of visit to health professional | **0.02** | **-.14** | **0.40** | **0.09** |
| Sleeping Duration | 0.01 | .11 | 1.97 | 0.17 |
| Sleeping Difficulties, (yes) | 0.01 | -.10 | 4.41 | 0.21 |
| Reason Sleep Difficulties (Ref: Foot pain) | 0.01 |  |  |  |
| Any other Pain |  | -.05 | 5.38 | 0.59 |
| Depression and anxiety |  | -.13 | 6.41 | 0.21 |
| Feeling Rested (Ref: Yes ) | 0.01 |  |  |  |
| Partially |  | -.04 | 5.30 | 0.69 |
| No |  | -.14 | 6.59 | 0.16 |
| Smoking (ref: never smoked) | 0.01 |  |  |  |
| Yes (Active, social smokers) |  | -.10 | 5.99 | 0.24 |
| No (Passive, ex-smokers) |  | .03 | 4.91 | 0.72 |
| Family History (Ref : None ) | 0.01 |  |  |  |
| Tendon disorders |  | -.01 | 7.16 | 0.92 |
| Psoriasis & Connective tissue disease |  | -.06 | 9.53 | 0.55 |
| Ankylosing spondylitis & RA |  | -.06 | 9.36 | 0.58 |
| Other |  | .06 | 16.22 | 0.46 |
| Psychological |  |  |  |  |
| PCS | **0.09** | **-.31** | **0.16** | **<0.001** |
| CSI | **0.10** | **-.31** | **0.12** | **<0.001** |
| FABQ-W | **0.03** | **-.18** | **0.22** | **0.03** |
| FABQ-PA | 0.01 | -.11 | 0.39 | 0.19 |
| Depression |  |  |  |  |
| Condition Prediction (ref: don’t know) | 0.05 |  |  |  |
| Get better |  | .11 | 5.08 | 0.26 |
| No change |  | .01 | 6.77 | 0.91 |
| Get worse |  | **-.17** | **8.54** | **0.06** |
| SOCIAL |  |  |  |  |
| Education (ref: PhD and Msc) | 0.07 |  |  |  |
| Bachelor |  | .09 | 5.43 | 0.37 |
| High school |  | .03 | 6.57 | 0.76 |
| Elementary school |  | **-.22** | **7.60** | **0.02** |
| Occupation (ref: Unemployment, students, homemakers , retired) | 0.02 |  |  | **0.01** |
| Blue-collar |  | .10 | **7.60** | 0.26 |
| White collar & Professionals |  | .16 | **5.05** | **0.09** |
| eHealth | 0.001 | -.08 | 0.31 | 0.35 |
| ACTIVITY |  |  |  |  |
| GPAQ | 0.002 | -.04 | 0.01 | 0.61 |
| Hours Standing | 0.002 | -.04 | 0.59 | 0.58 |
| Footwear | **0.02** | **-.15** | **0.08** | **0.07** |
| Sport Participation | **0.03** | **.19** | **4.36** | **0.02** |

**4. Univariate analyses results for logistic regression**

Supplementary table 4: Univariate analyses for logistic regression by comparing people with PHP (n=135) and people with other foot pain (n=99). The dependent variable is having PHP versus having other foot and ankle related musculoskeletal conditions. Odd ratio were the likelihood of having PHP, meaning greater than 1 increases the possibility of having PHP, while less than 1 decreases the possibility of having PHP. Key: mins= minutes, CI: Confidence Interval, Std= standard. ‡Handled as continuous in the models using the combined categories, assuming linearity and the coefficients are per category increase. Bold variables are indicating statistically significant results.

| Potential Predictors | Odds ratio | Std. Error | Confidence int. | P > I t I |
| --- | --- | --- | --- | --- |
| Increased Quality of Life | **1.02**  **15** | **0.007** | **1.00 – 1.03** | **0.004** |
| Demographics |  |  |  |  |
| Age | **1.03** | **.01** | **1.01 – 1.06** | **0.001** |
| BMI | **1.09** | **.03** | **1.02 – 1.16** | **0.005** |
| Sex (ref: male) | **1.62** | **.43** | **.95 – 2.76** | **0.07** |
| Ethnicity (ref: others and PNTS) | 0.008 |  |  |  |
| White | **2.11** | **.91** | **.90 – 4.93** | **0.08** |
| Asian | 1.64 | .52 | .87 – 3.06 | 0.11 |
| Biomedical |  |  |  |  |
| Morning Pain duration, mins. | 1.00 | .002 | 0.99 – 1.00 | 0.31 |
| Morning Pain Severity, VAS | **1.02** | **.004** | **1.01 – 1.03** | **<0.001** |
| Disease duration^‡^ | 1.05 | .006 | 0.94 – 1.18 | 0.33 |
| Comorbidity (ref: none) | 0.07 |  |  |  |
| Musculoskeletal d. |  | -.25 | 6.65 | 0.005 |
| Systemic d. |  | .04 | 5.39 | 0.64 |
| Number of Comorbidity | **2.02** | **.54** | **1.20 – 3.43** | **0.008** |
| Back pain presence, (ref: no) |  |  |  |  |
| Yes (Current, recurrent) | 1.54 | .55 | 0.76 – 3.11 | 0.22 |
| Yes (Previously) | **1.88** | **.61** | **1.00 – 3.55** | **0.04** |
| Pain in walking (ref: no change) |  |  |  |  |
| Worse | **0.51** | **-.15** | **.28 – .91** | **0.02** |
| Better | **0.24** | **-.10** | **.10 – .55** | **0.001** |
| Pain in standing (ref: no change) |  |  |  |  |
| Worse | **.33** | **.17** | **.12 - .93** | **0.03** |
| Better | **.31** | **.09** | **.17 - .56** | **<0.001** |
| Pain in sitting (ref: no change) |  |  |  |  |
| Worse | **.44** | **.15** | **.22 - .89** | **0.02** |
| Better | **.38** | **.15** | **.17 - .84** | **0.01** |
| Having previous injury (ref: yes) | **1.57** | **.43** | **.92 - 2.70** | **0.09** |
| Number of investigations | **1.34** | **.20** | **.99 – 1.81** | **0.05** |
| Number of visit to health professional | **1.04** | **.02** | **.99 – 1.09** | **0.09** |
| Sleeping Duration | **0.77** | **.09** | **.60 – .99** | **0.04** |
| Sleeping Difficulties, (yes) | **2.48** | **.73** | **1.39 – 4.43** | **0.002** |
| Feeling Rested (Ref: Yes ) |  |  |  |  |
| Partially | 1.52 | .46 | .84 – 2.76 | 0.16 |
| No | **2.38** | **1.01** | **1.04 – 5.47** | **0.04** |
| Smoking (ref: never smoked) |  |  |  |  |
| Yes (Active, social smokers) | 1.25 | .50 | .57 – 2.74 | 0.57 |
| No (Passive, ex-smokers) | .76 | .27 | .37 – 1.55 | 0.45 |
| Psychological |  |  |  |  |
| PCS | **1.04** | **.01** | **1.01 – 1.07** | **0.001** |
| CSI | **1.02** | **.009** | **1.01 – 1.04** | **0.009** |
| FABQ-W | **1.04** | **.01** | **1.01 – 1.07** | **0.007** |
| FABQ-PA | **1.08** | **.02** | **1.03 – 1.14** | **0.001** |
| Depression | 1.66 | .92 | 0.56 – 4.96 | 0.35 |
| Condition Prediction (ref: don’t know) |  |  |  |  |
| Get better | .58 | .19 | 0.30 – 1.13 | 0.11 |
| No change | .63 | .27 | 0.27 – 1.49 | 0.30 |
| Get worse | .48 | .24 | 0.17 – 1.32 | 0.15 |
| SOCIAL |  |  |  |  |
| Education (ref: PhD and Msc) |  |  |  |  |
| Bachelor | 1.2 | .38 | 0.64 – 2.24 | 0.56 |
| High school | 1.67 | .69 | 0.74 – 3.77 | 0.21 |
| Elementary school | 3.87 | 2.38 | 0.15 – 12.91 | 0.20 |
| Occupation (ref: Unemployment, students, homemakers , retired) |  |  |  |  |
| White collar & Professionals | 0.76 | .42 | 0.25 – 2.29 | 0.62 |
| Blue-collar | **0.34** | **.12** | **0.16 – .71** | **0.004** |
| eHealth | 1.01 | .01 | 0.97 – 1.05 | 0.40 |
| ACTIVITY |  |  |  |  |
| GPAQ | 0.99 | 0.000 | 0.99-1.00 | 0.36 |
| Hours Standing | 1.01 | 0.03 | 0.94 – 1.09 | 0.92 |
| Footwear | **1.01** | **0.005** | **1.00 – 1.02** | **0.01** |
| Sport Participation | **0.39** | **0.11** | **0.23 – 0.62** | **0.001** |
